# Public Health Data Applications Using the CDC Tracking Network: Augmenting Environmental Hazard Information With Lower‐Latency NASA Data

**DOI:** 10.1029/2023GH000971

**Published:** 2023-12-13

**Authors:** H. M. Amos, N. K. Skaff, S. Schollaert Uz, F. S. Policelli, D. Slayback, E. Macorps, M. J. Jo, K. Patel, C. A. Keller, P. Abue, V. Buchard, A. K. Werner

**Affiliations:** ^1^ Earth Science Division Goddard Space Flight Center National Aeronautics and Space Administration Greenbelt MD USA; ^2^ Science Systems and Applications, Inc. Lanham MD USA; ^3^ National Center for Environmental Health Centers for Disease Control and Prevention Atlanta GA USA; ^4^ NASA Postdoctoral Program, NASA Goddard Space Flight Center Greenbelt MD USA; ^5^ University of Maryland Baltimore County Baltimore MD USA; ^6^ University of Texas Austin TX USA; ^7^ Morgan State University Baltimore MD USA

**Keywords:** air quality, flood, environmental public health, remote sensing

## Abstract

Exposure to environmental hazards is an important determinant of health, and the frequency and severity of exposures is expected to be impacted by climate change. Through a partnership with the U.S. National Aeronautics and Space Administration, the U.S. Centers for Disease Control and Prevention's National Environmental Public Health Tracking Network is integrating timely observations and model data of priority environmental hazards into its publicly accessible Data Explorer (https://ephtracking.cdc.gov/DataExplorer/). Newly integrated data sets over the contiguous U.S. (CONUS) include: daily 5‐day forecasts of air quality based on the Goddard Earth Observing System Composition Forecast, daily historical (1980‐present) concentrations of speciated PM_2.5_ based on the modern era retrospective analysis for research and applications, version 2, and Moderate Resolution Imaging Spectroradiometer (MODIS) daily near real‐time maps of flooding (MCDWD). Data integrated into the CDC Tracking Network are broadly intended to improve community health through action by informing both research and early warning activities, including (a) describing temporal and spatial trends in disease and potential environmental exposures, (b) identifying populations most affected, (c) generating hypotheses about associations between health and environmental exposures, and (d) developing, guiding, and assessing environmental public health policies and interventions aimed at reducing or eliminating health outcomes associated with environmental factors.

## Key Points

- Timely information can be critical for public health decision making, but environmental hazard data can be fragmented or hard to use
- This work brings timely, routinely updated National Aeronautics and Space Administration (NASA) data into CDC Tracking Network's Data Explorer in familiar and accessible formats
- For CONUS, daily NASA air quality forecasts, historical air quality data, and daily flood maps are integrated into the Data Explorer

## 1 Introduction

Harmful environmental exposures are a significant contributor to morbidity and mortality in the U.S. and numerous health outcomes are expected to worsen under climate change (USGCRP, 2016; Watts et al., 2021). The U.S. National Aeronautics and Space Administration uses satellites, aircraft, ground measurements, and Earth system models to study the long‐term state of the environment as well as produce near real‐time (NRT) data about environmental hazards such as natural disasters (NASA, 2023c). The U.S. Centers for Disease Control and Prevention's (CDC) National Environmental Public Health Tracking Program (CDC Tracking Program) tracks exposures and health effects associated with environmental hazards (CDC, 2023b). The Environmental Public Health Tracking Network (CDC Tracking Network), built by the CDC Tracking Program, is a dynamic, web‐based system that brings together health data and environmental data from a network of partners to provide timely, relevant information for better community health (https://ephtracking.cdc.gov/). This paper describes recent efforts to augment information provided through the CDC Tracking Network with lower‐latency, higher spatiotemporal resolution NASA data sets for high priority environmental hazards. The overall goal is to enable health researchers and practitioners to access critical environmental data needed to understand and respond to health risks and make data‐informed decisions.

The mission of the CDC Tracking Program is to provide information from a nationwide network of integrated health and environmental data that drives actions to improve community health (CDC, 2023b). The CDC Tracking Network is a multi‐tiered, web‐based system with components at national, state, and local levels that unifies health and environmental data from a network of varied sources and makes that information publicly available to its audience of public health researchers, professionals, decision makers, and the public in standardized formats (CDC, 2023b). In collaboration with federal, state, and local partners, priority environmental health issues and key surveillance questions are identified. Existing data are evaluated for their ability to inform these issues and then integrated into the CDC Tracking Network. Data are disseminated through the CDC Tracking Network's flagship product, the Data Explorer (https://ephtracking.cdc.gov/DataExplorer/), as well as dashboards, infographics, and an application programming interface (API). Additionally, when gaps or new data needs are identified, the CDC Tracking Program collaborates with partners (https://www.cdc.gov/nceh/tracking/partners) to develop standards for data collection, develop models, expand the utility of non‐traditional public health data, or develop new methodologies for using existing data.

Near real‐time monitoring and forecasting of environmental hazards have the potential to reduce harmful exposures by enabling early warning systems and other timely public health interventions (e.g., WMO, 2023). Currently, critical NRT data may not be readily accessible to public health practitioners, particularly those that need to react to public health emergencies, because the data are fragmented across multiple data systems and are not formatted in a manner consistent with typical public health uses (e.g., using administrative boundaries such as county and census tract) (Duncan et al., 2021; Liu et al., 2021). Augmenting and improving data pipelines and processes to host these data in a usable and standardized format and disseminating these data via the CDC Tracking Network will assist in making important information about environmental hazards more readily accessible to public health practitioners and partners, including relevant CDC programs, and state, tribal, local, and territorial health departments.

The objective of this work is to leverage the NASA Goddard Space Flight Center (GSFC) expertise in processing and interpreting a wide range of environmental satellite data products and to make these data more accessible to public health practitioners through the CDC Tracking Network. Section 2 describes the data needs of the CDC Tracking Program and efforts to identify environmental data sets by NASA GSFC to meet these needs. Section 3 details the data systems methodology and implementation processes to define and establish routine workflows for transforming and transferring data in an automated and timely fashion. Section 4 illustrates applications of the data. Section 5 summarizes limitations of the data sets and offers some guidance on appropriate uses in public health context. Sections 6 and 7 capture outlooks and conclusions, respectively.

## 2 Public Health Environmental Data Needs

This section describes the collaborative process between the CDC Tracking Program and NASA GSFC around the use of available environmental hazard data to address current CDC data needs. Priority topic areas include air quality, flooding, extreme weather, wildfires, and climate impacts. These priorities reflect gaps in existing content on the CDC Tracking Network's Data Explorer, feedback from the CDC Tracking Network's Content Work Group focused on air quality, climate, and weather as areas of programmatic emphasis within the CDC National Center for Environmental Health and the CDC Division of Environmental Health Science and Practice. The CDC Tracking Program identified a need for lower latency data (i.e., shorter lag time between data production and availability to users) because of the potential to inform public health emergencies and to have timelier data for decision‐making. This paper presents progress on the first two priority topic areas of air quality and flooding.

### 2.1 Data Selection

NASA GSFC produces numerous publicly available data products relevant for air quality (https://airquality.gsfc.nasa.gov/) and flooding (NASA, 2023b). Product applicability was assessed based on geographic coverage and resolution, temporal coverage and resolution, latency, means of data access, file formats, and levels of processing (e.g., Level 1–Level 5 (NASA, 2023a)). Broadly, common features of the selected data are that they are available daily over CONUS, remotely accessible through an API, and produced on an ongoing basis. The suitable data products selected are summarized in Table 1.

**Table 1:** National Aeronautics and Space Administration Goddard Research‐Grade Products for Air Quality and Flooding Implemented Into the CDC Tracking Program's Workflow

Data product	Resolution	Strengths and limitations for public health uses
GEOS‐CF air quality forecast	Daily 5‐day forecast	+ daily forecast of criteria pollutants
	25 km × 25 km grid, global	− coarse resolution
MERRA‐2 aerosols	Daily, 1980‐present	+40‐year record, daily resolution
	50 km × 50 km, global	− coarse resolution, 1‐month lag
MODIS NRT global daily flood product (MCDWD)	Daily, 2022‐present	+ low latency, daily coverage
	250 m, global	− cannot see through clouds

### 2.2 Air Quality

The air quality data products selected are the Goddard Earth Observing System Composition Forecast (GEOS‐CF, https://gmao.gsfc.nasa.gov/weather_prediction/GEOS-CF/) and the Modern‐Era Retrospective analysis for Research and Applications, Version 2 (modern era retrospective analysis for research and applications, version 2 (MERRA‐2), https://gmao.gsfc.nasa.gov/reanalysis/MERRA-2/). Both are global gridded products made publicly available by the NASA GSFC Global Modeling and Assimilation Office (GMAO) on an ongoing basis. GEOS‐CF is a global three‐dimensional model of atmospheric composition generated using the GEOS Earth System Model coupled with the GEOS‐Chem chemical transport model, with initial meteorological conditions constrained by satellite observations (Keller et al., 2021). Anthropogenic air pollutant emissions are obtained from pre‐defined emission inventories (Crippa et al., 2018) and wildfire emissions are derived daily from satellite observations using the Quick Fire Emissions Data set (quick fire emissions dataset) (Darmenov & da Silva, 2015). GEOS‐CF produces daily 5‐day global forecast at approximately 25 km × 25 km horizontal resolution of surface concentrations of criteria pollutants designated by the U.S. Environmental Protection Agency (USEPA, 2023): particulate matter less than 2.5 µm in diameter (PM_2.5_), surface ozone (O_3_), nitrogen dioxide (NO_2_), sulfur dioxide (SO_2_), and carbon monoxide (CO) (Keller et al., 2021; Knowland et al., 2022). GEOS‐CF contributes a novel forecasting capability to the suite of air quality information currently made available on the CDC Tracking Network. MERRA‐2 is an atmospheric re‐analysis product fusing measurements and model results to produce a physically consistent estimate of the state of the atmosphere and aerosols for the period 1980‐present at approximately 50 km × 50 km horizontal resolution with a latency of approximately 1 month behind real‐time (Bosilovich et al., 2015; Buchard et al., 2016, 2017; Gelaro et al., 2017; Randles et al., 2016, 2017). MERRA‐2 uniquely contributes a long (+42 years) historical PM_2.5_ record and detailed information about the aerosol constituents (e.g., black carbon, organic carbon) of total PM_2.5_.

### 2.3 Flooding

**Figure 1:**
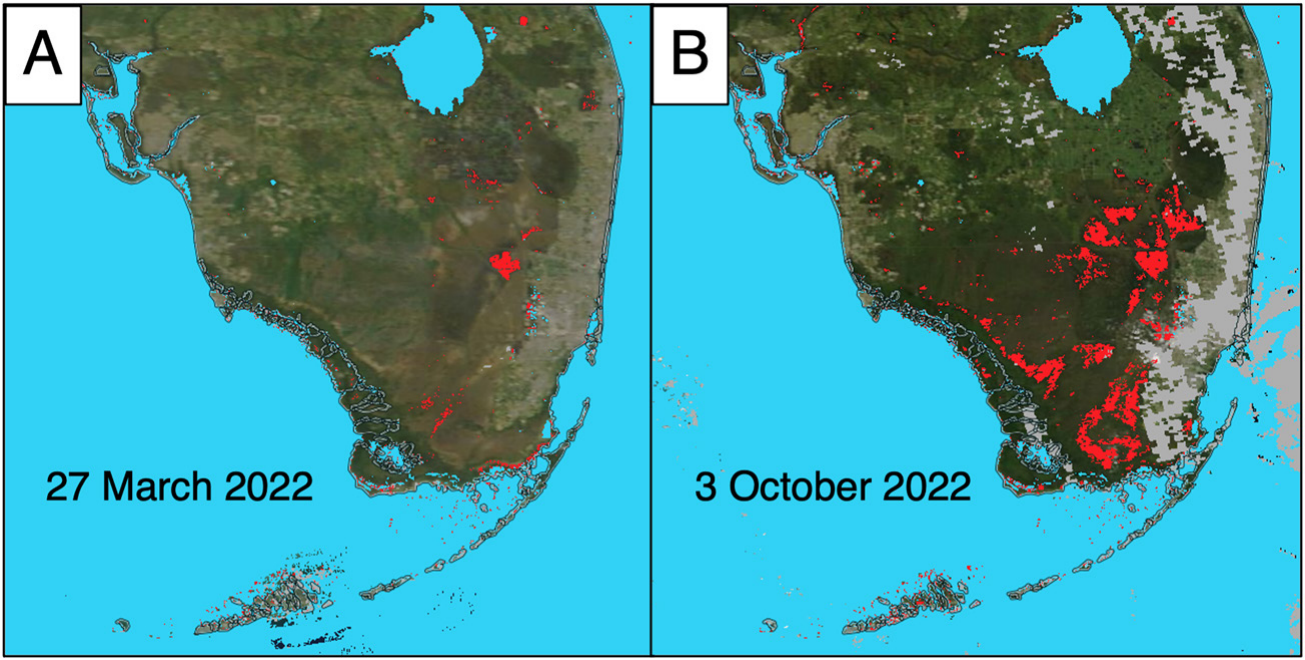
Flooding before (a) and after (b) Hurricane Ian as mapped by the National Aeronautics and Space Administration MODIS near real‐time global flood product (MCDWD). Flooded pixels are red, gray pixels are clouds. Ian made landfall on 28 September 2022 near Fort Meyers, Florida as a Category 4 hurricane. (a) 27 March 2022 is shown to illustrate a representative baseline of conditions before hurricane season. (b) Not all the flood water detected on 3 October 2022 is from Hurricane Ian. Flood water can persist for weeks or longer.

The flood product selected is the Moderate Resolution Imaging Spectrometer (MODIS) NRT (within 3–5 hr after observation) global flood product (MCDWD) (LANCE MCDWD, 2022). MCDWD is a publicly available satellite‐based data product that estimates the presence and surface extent of flood waters (i.e., standing water) over land (Figure 1). MCDWD is provided at ∼250‐m resolution, with near‐global daily coverage spanning 2011‐present and is derived from the MODIS Surface Reflectance (MOD09) data sets from the Aqua and Terra satellites (NASA, 2023d, k; Slayback, 2023]. MCDWD is processed and made publicly available within 3 hr of collection by NASA's Land, Atmosphere NRT Capability for EOS (LANCE, https://lance.modaps.eosdis.nasa.gov/). Detailed descriptions of the MCDWD product, algorithms, product evaluation, data access, and planned improvements are available in Slayback (2023) and online https://www.earthdata.nasa.gov/global-flood-product. Near real‐time high‐resolution (3‐m) flood maps derived from commercial PlanetScope imagery (Policelli, 2022) were considered but excluded because their application is presently more suited for limited areas of interest and routinely generating maps covering CONUS is not yet feasible.

## 3 Implementation and Data Systems Methodology

**Figure 2:**
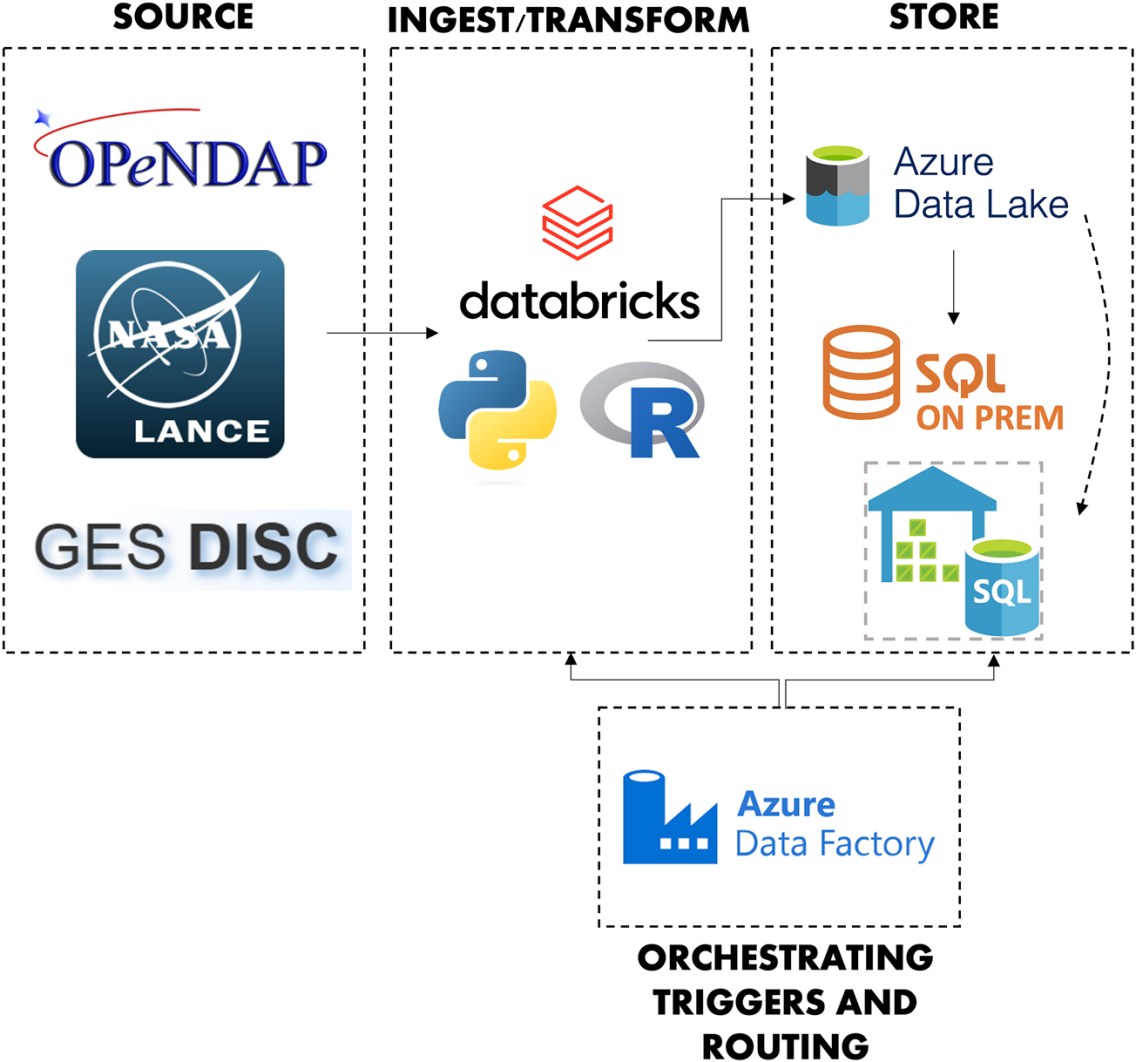
Schematic of the CDC Tracking Network's extract, transform, and load (ETL) workflow.

Through an interagency partnership, the goal is to define and establish routine processes for transforming and transferring NASA GSFC data to the CDC Tracking Program and its partners in an automated and timely fashion for dissemination via the CDC Tracking Network. Processes are developed in the CDC Enterprise Data Analytics and Visualization (EDAV) Platform, which is a cloud‐based data management and processing ecosystem where users can store, transform, and analyze data. EDAV is built primarily using Microsoft Azure cloud services. Azure's implementation of Databricks, a web‐based analytics platform, is used for scripting data ingestion from NASA APIs, further transformation of gridded raw data to geopolitical boundaries, and calculating relevant quantities (e.g., sum, mean, area) using Python and R. Completed data are routed to the Azure Data Lake, where most recent and archival versions of daily data runs are stored. Azure Data Factory is used to orchestrate the initialization of Databricks scripts in the appropriate order and the routing of data between different storage environments (Figure 2).

### 3.1 Air Quality

The air quality data products are queried remotely, spatially transformed from model grid to U.S. counties or census tracts, depending on the data set, and converted to measures tailored to environmental public health audiences. An automated daily query pulls GEOS‐CF via open‐source project for a network data access protocol, retrieving global 5‐day forecasts for PM_2.5_, O_3_, SO_2_, NO_2_, and CO. Forecast results are extracted for the surface (model level 1) for CONUS and transformed from the GEOS‐CF model grid to U.S. counties based on boundaries derived from Census Topologically Integrated Geographic Encoding and Reference system (TIGER) 2000 (NOAA, 2023) (see Section 6 for planned updates). Daily statistics (24‐hr mean [PM_2.5_, SO_2_], 24‐hr maximum [SO_2_, NO_2_], and maximum 8‐hr rolling mean [CO, O_3_]) are calculated from hourly values for each county. An automated monthly query pulls MERRA‐2 M2T1NXAER from the Goddard Earth Sciences Data and Information Services Center (GES DISC) (GMAO, 2015). Surface (model level 1) aerosol constituents, SO_2_, and CO concentrations are extracted for CONUS. PM_2.5_ concentrations in micrograms per cubic meter are estimated by summing the aerosol constituents following (Buchard et al., 2017).

$${\text{PM}}_{2.5}={\text{PM}}_{2.5}\left[\text{DU}\right]+{\text{PM}}_{2.5}\left[\text{SS}\right]+{\text{PM}}_{2.5}\left[\text{OC}\right]+{\text{PM}}_{2.5}\left[\text{BC}\right]+\left(132.14/96.06\right)*{\text{PM}}_{2.5}\left[{\text{SO}}_{4}\right]$$

where DU is dust, SS is sea salt, OC is organic carbon, BC is black carbon, SO_4_ is sulfate, and the factor of 132.14/96.06 is applied to convert sulfate ion (molar mass of 96.06 g mol^−1^) concentration output by MERRA‐2 to ammonium sulfate (132.14 g mol^−1^), assuming that sulfate is primarily present as neutralized ammonium sulfate. MERRA‐2 SO_2_ surface concentrations are converted from kilograms per cubic meter (kg m^−3^) to parts per billion (ppb) using the following equation:

$${\text{SO}}_{2}\left(\text{ppb}\right)=\left(T\left(k\right)*{\text{SO}}_{2}\;\left(\text{kg}\;m^{-3}\right)*8.314*10^{7}\right)/\left(1.013*64.066\right)$$

Coordinated Universal Times are converted to the local time of each county centroid, then daily statistics (24‐hr mean [PM_2.5_ (and constituents), SO_2_], 24‐hr maximum [SO_2_, NO_2_], and maximum 8‐hr rolling mean [CO]) are calculated from hourly values for each county. The Air Quality Index (AQI) and AQI category (reflecting level of health concern) associated with each daily concentration for both GEOS‐CF and MERRA‐2 estimates are determined as specified in the U.S. EPA technical documentation (USEPA, 2018). Concentrations higher than the AQI scale (“beyond the AQI”) are classified as if they are in the highest AQI category (USEPA, 2018).

**Figure 3:**
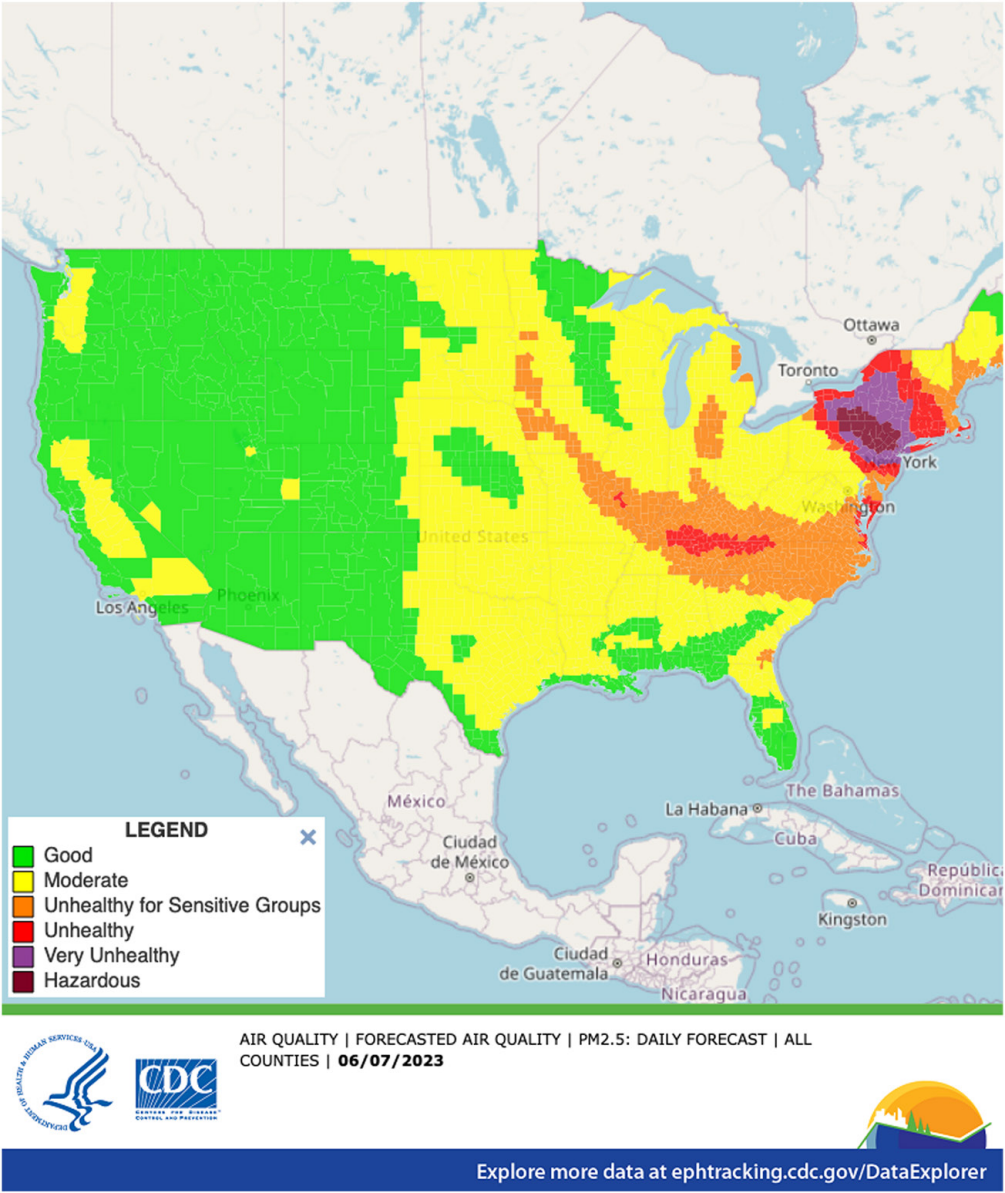
Example of a map of Goddard Earth Observing System Composition Forecast forecasted PM_2.5_ as seen on the CDC Tracking Network's publicly available Data Explorer. On this day, 6 June 2023, heavy smoke from Canadian wildfires impacted major population centers in the eastern U.S.

County‐level daily AQI category estimates for GEOS‐CF and MERRA‐2 air pollutant estimates are mapped on the CDC Tracking Network's Data Explorer (Figure 3). Measures incorporating GEOS‐CF data are housed within the “Forecasted Air Quality” indicator and those derived from MERRA‐2 can be found in the “Current and Historical Air Quality” indicator, both of which are in the “Air Quality” content area. The “Forecasted Air Quality” indicator relies entirely on GEOS‐CF data that updates each morning at approximately 7 a.m. U.S. Eastern Standard Time (EST) and includes a 1‐day hindcast and 4‐day forecast (including current day). MERRA‐2 is incorporated in a suite of daily air quality measures that also includes monitor data from the U.S. EPA's AirNow platform (https://www.airnow.gov/) (CDC, 2023a). These measures include data from the previous several months, though only AirNow data is available for the present month due to the 1‐month latency of MERRA‐2. Users can view the underlying concentration used to derive the AQI category by hovering the cursor over a county of interest. All AQI category and concentration data are also available in tabular format using the data download tool on the Data Explorer or via the CDC Tracking Network API.

### 3.2 Flooding

The MCDWD flood product is used to generate the “Total Area Flooded” and “Total Population Affected by Flooding” measures on the CDC Tracking Network's Data Explorer. Measures incorporating MCDWD data are housed within the “Current and Historical Flooding” indicators in the “Precipitation & Flooding” content area. The data are queried remotely, mosaicked, clipped, and spatially summarized from raster data to U.S. counties, U.S. territory county equivalents, and census tracts. A daily automated query retrieves MCDWD from the NASA LANCE system (https://lance.modaps.eosdis.nasa.gov/). The query is made at 3 a.m. EST to retrieve the most‐recent 2‐day composite. The data are provided by LANCE in 10° × 10° tiles, with variables for two types of flood conditions: recurring flooding (available in 2024, see Section 6) and unusual flooding. The total area flooded is estimated by determining the proportion of the MCDWD tiles where flood has been identified relative to the total raster overlap with each jurisdiction. This proportion is then multiplied by the total area of the jurisdiction. Separate calculations are made to determine the quantity of recurring and unusually flooded land. To estimate the total number of people affected by flooding within each county and census tract, the sum of the population of all the affected census blocks within each county and tract is calculated. A block is considered affected if a flooded tile is identified anywhere within its boundary. Similarly, separate calculations are made to determine the total population affected by recurring and unusual flooding.

## 4 Applied Uses

Data integrated into the CDC Tracking Network are broadly intended to inform both research and early warning activities, including (a) describing temporal and spatial trends in disease and potential environmental exposures, (b) identifying populations most affected, (c) generating hypotheses about associations between health and environmental exposures, and (d) developing, guiding, and assessing environmental public health policies and interventions aimed at reducing or eliminating health outcomes associated with environmental factors. Numerous contextual data are available on the Data Explorer that can be viewed contemporaneously to help drive data‐informed decision‐making, such as biomonitoring data, disease burdens, community characteristics, and environmental justice indicators. Hypothetical use cases are provided below for illustrative purposes.

- *Target public health surveillance*: In the CDC Tracking Network's Data Explorer, the “Total Area Flooded” measure derived from MCDWD can provide situational awareness and identify where to target public health surveillance, community services, environmental sampling, or additional imagery acquisition (e.g., drone, commercial satellite). The maps can assist in identifying inundated areas, as well as dry areas where emergency response assets can be staged. The Data Explorer also has points of interest, such as nursing homes and day care centers, that can be viewed alongside a map of “Total Area Flooded” to better inform public health activities. Environmental justice measures, such as populations that are linguistically isolated, can also be layered on the Data Explorer map to help prioritize assistance.
- *Reduce personal exposure*: In summer 2023, a historic Canadian wildfire smoke event left millions of residents in the northeastern U.S. under air quality advisories (NASA, 2023e). During a similar major air quality, an individual with asthma might consult the “Forecasted Air Quality” indicator derived from GEOS‐CF on the CDC Tracking Network's Data Explorer alongside AirNow and other sources of information to inform the timing and confidence around decisions of when (or if) to perform strenuous exercise outdoors.
- *Examine trends in patient‐centered outcomes*: A longitudinal study of wildfire smoke impacts on patient‐centered outcomes for adult patients with asthma might examine the “Current and Historical Air Quality” indicators derived from MERRA‐2 on the CDC Tracking Network's Data Explorer for insight on spatiotemporal trends in smoke exposure, cumulative exposures, timing and duration of smoke events, as well as statistical anomalies. In the Data Explorer, measures of PM_2.5_ and its constituents can be viewed alongside indicators of social vulnerability, occupation, and environmental justice indices to illuminate the interplay of wildfire smoke exposure and social determinants of health. The study results might then inform improved interventions such as refined public health messaging to populations who are at higher risk for asthma‐related emergency department visits or hospitalizations during wildfire events.

## 5 Limitations

### 5.1 Air Quality

The GEOS‐CF 5‐day air quality forecast and MERRA‐2 historical re‐analysis provide regional estimates of air quality. Greater caution is warranted when interpreting results in geographic areas with strong pollution gradients (e.g., urban areas), mountainous terrain, locations near emission point sources, and locations on the coast. On average, GEOS‐CF and MERRA‐2 have greater skill in the eastern U.S. and in rural areas (Buchard et al., 2017; Randles et al., 2016). MERRA‐2 has less skill during winter and spring and in environments dominated by nitrate aerosol (e.g., intense agricultural areas) because nitrate is not explicitly accounted for in the GOCART aerosol mechanism (Randles et al., 2016). MERRA‐2 is constrained by aerosol optical depth, and there is inherent uncertainty in how aerosols are vertically distributed in the model. For GEOS‐CF, forecast skill is generally greater for the nearer‐term forecasts (i.e., Day 1, Day 2) (Keller et al., 2021). For both GEOS‐CF and MERRA‐2, relative changes and trends are more robust than absolute changes. The air quality measures displayed in the Data Explorer represent outdoor ambient air pollution concentrations and do not directly reflect human exposures. The relationships between outdoor ambient concentrations, indoor air pollution, and individual exposures are active subjects of ongoing research, and likely vary depending upon pollutant, behavioral patterns, microenvironments, and building ventilation (Özkaynak et al., 2009; Patel et al., 2020; Singer et al., 2020). The NASA air quality data products disseminated via the CDC Tracking Network are research‐grade products and should not be used to assess National Ambient Air Quality Standards compliance or to evaluate progress toward attaining compliance.

### 5.2 Flooding

MCDWD provides estimates of surface flood water extent at the time of satellite overpass between roughly 70N–70S. Greater caution is warranted in interpreting results for MODIS data under shadows caused by terrain and clouds, which can strongly resemble water in the spectral bands (red and near infra‐red) used to generate the product (Slayback, 2023). Flash floods have a low likelihood of being observed due to their rapid appearance and disappearance, unless this coincides with the twice‐daily observation times of the Terra and Aqua satellites at approximately 10:30 a.m. and 1:30 p.m. local solar time (Gosset et al., 2023). Flooding in urban areas is also difficult for the 250‐m resolution MCDWD product to detect, due to the mismatch in sensor resolution to on‐the‐ground flooded areas. In addition to shadows, dark volcanic rock and recently melted snow can result in false positives, and vegetation cover can result in false negatives in MCDWD. The MODIS instruments aboard Terra and Aqua have flown in space for over 20 years and these missions have been scheduled for decommissioning in the 2025–2026 timeframe. MCDWD is transitioning to ingest data from the Visible Infrared Imaging Radiometer Suite (VIIRS) aboard the joint NASA/National Oceanic and Atmospheric Administration (NOAA) Suomi National Polar‐orbiting Partnership (Suomi‐NPP) and NOAA‐20 satellites.

## 6 Future Planned Work

Planned air quality‐related updates include: improving the computational efficiency and flexibility of the Python workflow used to spatially aggregate MERRA‐2 and GEOS‐CF data; adding coverage over Alaska, Hawaii, Puerto Rico, and Virgin Islands; and updating the county and census boundary shapes to Census TIGER 2020 (Census, 2021). The update to Census TIGER 2020 boundaries is anticipated to mostly impact Alaska and Bedford, Virginia.

Planned flooding updates include: MCDWD recurring flood classification and exploring a second, complimentary flood product. The NASA MCDWD team plan to update the product to include a flood classification for “recurring flood” (water occurring where it has occurred in the past with some regularity, but is not permanent water) in early 2024. Maps on the CDC Tracking Network's Data Explorer will be updated with new data when available. The HydroSAR flood product is being explored to potentially augment MCDWD daily maps. HydroSAR HyP3‐watermap is a 30‐m surface water extent product with a 12‐day revisit period based on European Space Agency's Sentinel‐1 C‐band synthetic aperture radar (SAR) (ASF, 2023). The HydroSAR project focuses on cloud‐based SAR data processing for rapid response and mapping of hydrological hazards. HydroSAR maps are retrieved by querying Alaska Satellite Facility’s (ASF) Hybrid Pluggable Processing Pipeline service (Hogenson et al., 2020). HydroSAR's revisit time is expected to be reduced to near‐daily coverage with the planned launches of ESA's Sentinel‐1C and NASA‐Indian Space Research Organization (Indian Space Research Organization) SAR (NISAR) in 2024. For public health surveillance and practice, the unique contributions of HydroSAR would be an order of magnitude increase in spatial resolution (MCDWD 250‐m vs. HydroSAR 30‐m), and SAR technology has all‐weather and day‐and‐night imaging capabilities.

## 7 Conclusions

Through a partnership between the CDC Tracking Program and NASA GSFC that was established in 2022, critical environmental data products are delivered into the CDC Tracking Network's nationwide network of integrated health and environmental data in order to drive actions to improve community health. Near real‐time monitoring and forecasting of environmental hazards have the potential to reduce harmful exposures by enabling early warning systems and other timely public health interventions. Currently, NRT NASA data may not be readily accessible to public health practitioners, particularly those that need to react to public health emergencies, because the data are fragmented across multiple non‐interoperable data systems and are not formatted in a manner consistent with typical public health uses. Developing data pipelines and processes to host these data in a usable and standardized format and disseminating these data via the CDC Tracking Network assists in making important information about environmental health hazards more readily accessible to public health practitioners, decision makers, and partners.

Based on CDC and partner priorities, NASA GEOS‐CF daily 5‐day air quality forecasts, MERRA‐2 historical daily PM_2.5_ concentrations, and MCDWD daily NRT maps of flooding are integrated into the CDC Tracking Network's publicly accessible Data Explorer (https://ephtracking.cdc.gov/DataExplorer/). These data products are mature, well‐established resources produced on an ongoing basis and can be remotely accessed with automated queries to deliver timely CONUS‐wide maps of environmental hazards. With the forthcoming decommissioning of the Aqua/Terra satellites, MCDWD will transition to ingesting VIIRS/Suomi NPP observations to ensure continuity. Augmenting MCDWD flood maps with other satellite‐based flooding data products is being explored. The air quality and flooding data products newly integrated into the CDC Tracking Network are broadly intended to promote improve community health through data‐informed decisions by informing trends, identifying potentially affected populations, and informing interventions that support better patient‐centered outcomes.

## Disclaimer

The findings and conclusions in this report are those of the authors and do not necessarily represent the official position of the Centers for Disease Control and Prevention or National Aeronautics and Space Administration.

## Conflict of Interest

The authors declare no conflicts of interest relevant to this study.

## Data Availability

All data sources in this manuscript are free and publicly available. NASA GEOS‐CF 5‐day forecasts of air quality pollutants are accessed via OpenDAP (GMAO, 2023; Keller et al., 2021). NASA MERRA‐2 historical aerosol concentrations are accessed via GES DISC (GMAO, 2015). The NASA MCDWD daily NRT flood product is accessed via LANCE (LANCE MCDWD, 2022). Open‐source software is used to query the data, spatially transform the data, and compute measures consumable to a public health audience (e.g., AQI). The code is written in R (R Foundation, 2022) and Python (Python Software Foundation, 2021). The air quality and flooding measures are available as CONUS‐wide maps and as downloadable data layers on the CDC Environmental Public Health Tracking Network Data Explorer (CDC, 2023b).
